# Supplementary material for: Epimeric Mixture Analysis and Absolute Configuration Determination Using an Integrated Spectroscopic and Computational Approach—A Case Study of Two Epimers of 6-Hydroxyhippeastidine
Source: Molecules. 2022 Dec 26;28(1):214. doi: 10.3390/molecules28010214 (PMC9822407; doi:10.3390/molecules28010214)
Supplement: Supplementary file 1 [file molecules-28-00214-s001.zip › molecules-2011734-supplementary.pdf]

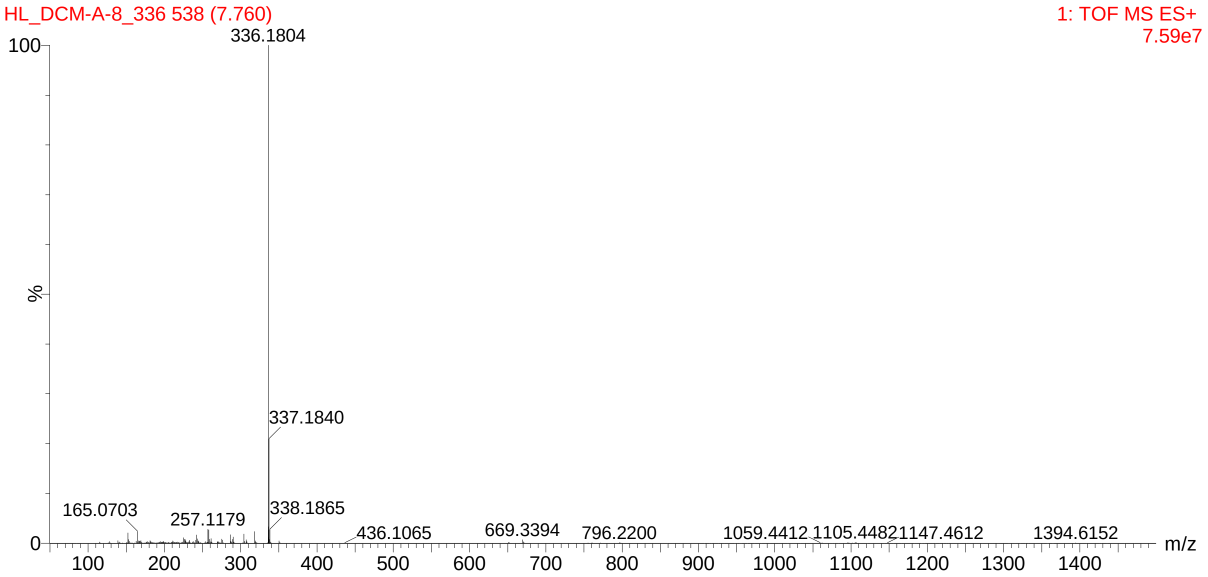


**Figure S1.** HRMS spectrum of compounds 1 and 2.

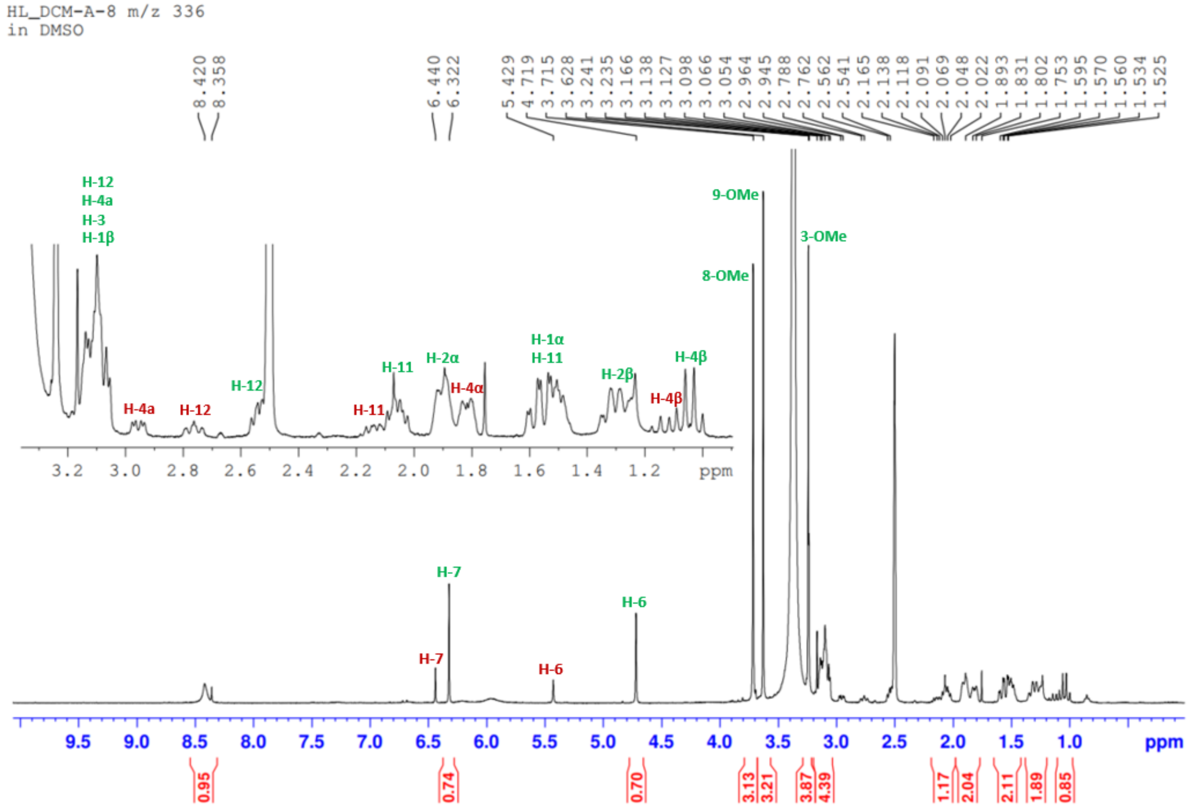


**Figure S2**. $^{1}$H spectrum of compounds **1** and **2** in $(CD_3)_2SO$ (minor epimer, major epimer and overlapping signals).

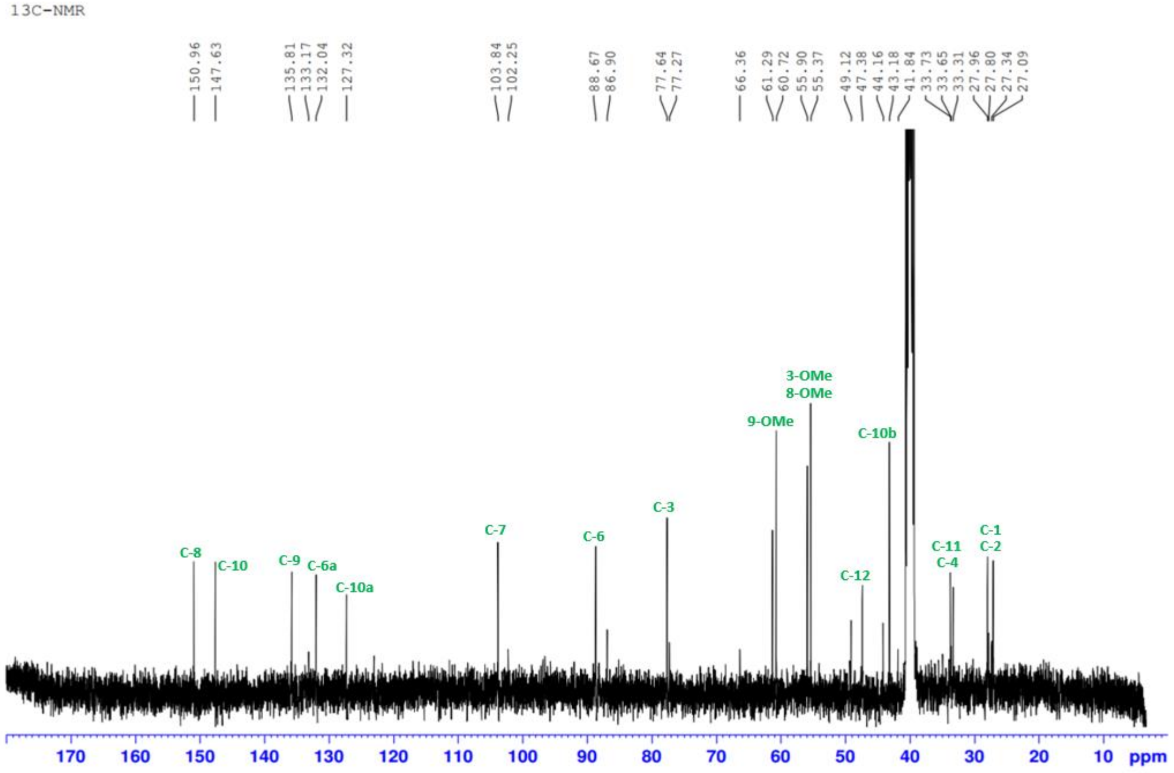


**Figure S3.** $^{13}C$ spectrum of compounds **1** and **2** in $(CD_3)_2SO$. Carbon signals of the major epimer was labelled.

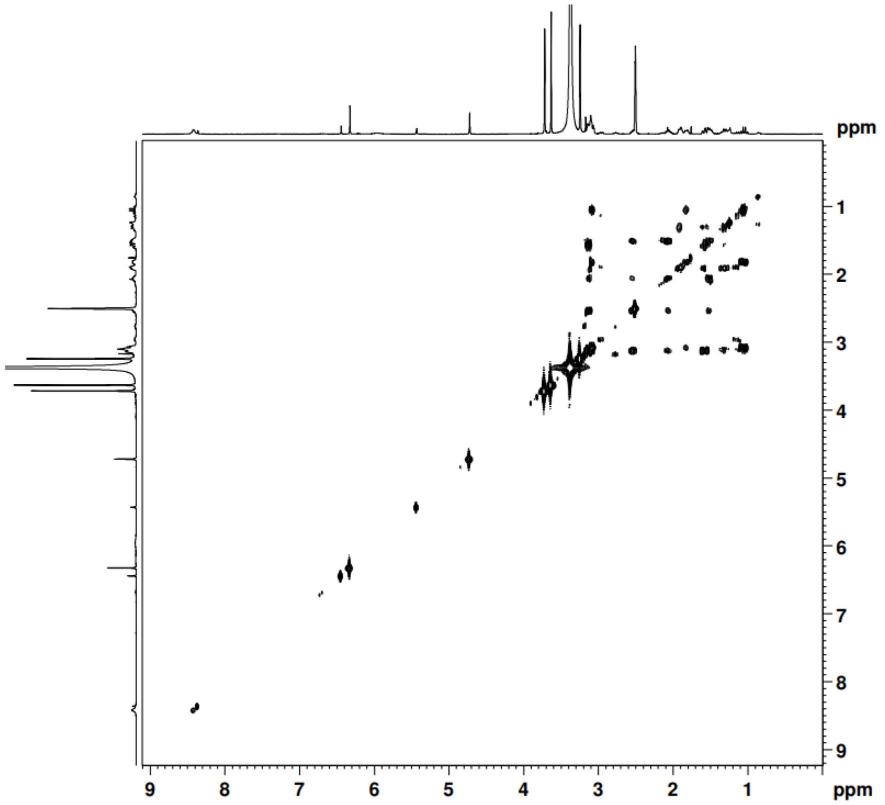


**Figure S4.** COSY spectrum of compounds **1** and **2** in $(CD_3)_2SO$.

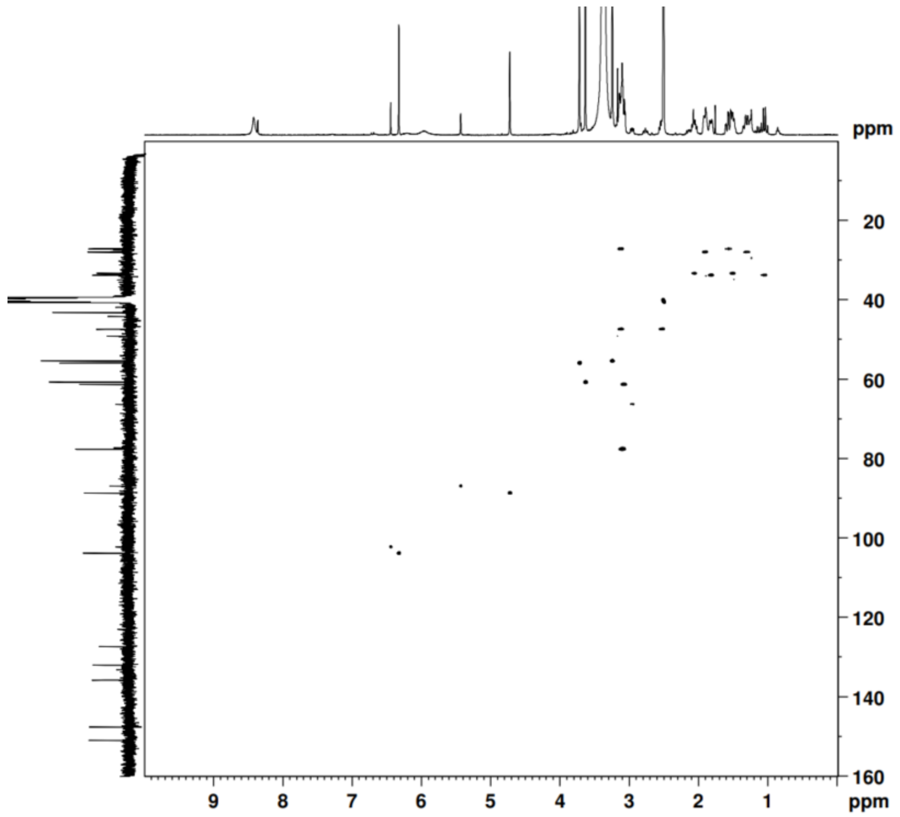


**Figure S5.** HSQC spectrum of compounds **1** and **2** in $(CD_3)_2SO$.

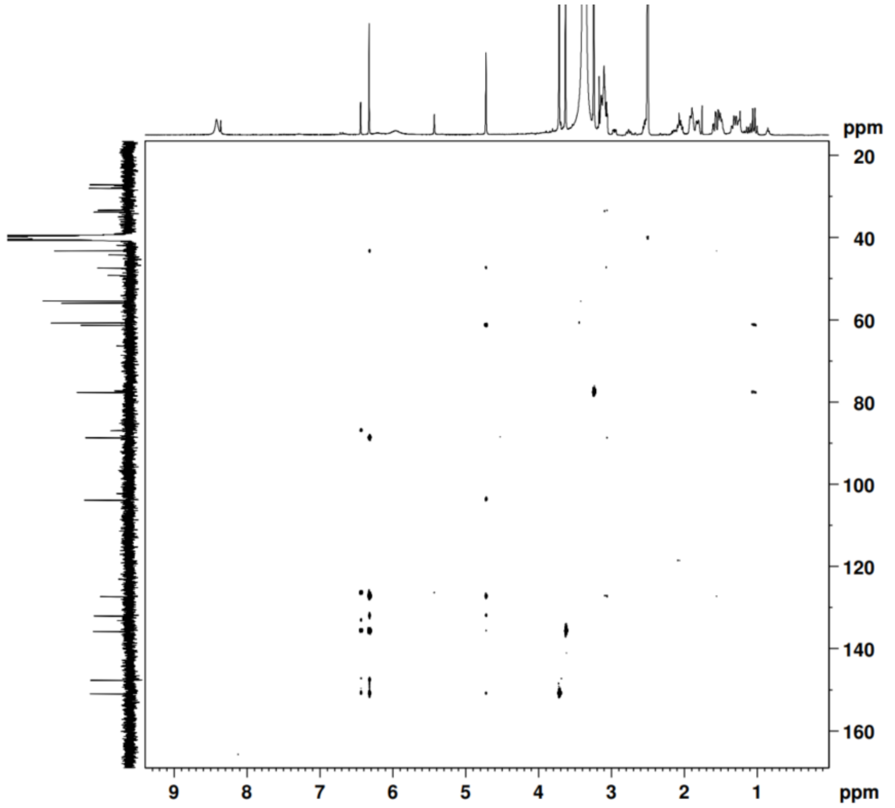


**Figure S6.** HMBC spectrum of compounds **1** and **2** in $(CD_3)_2SO$.

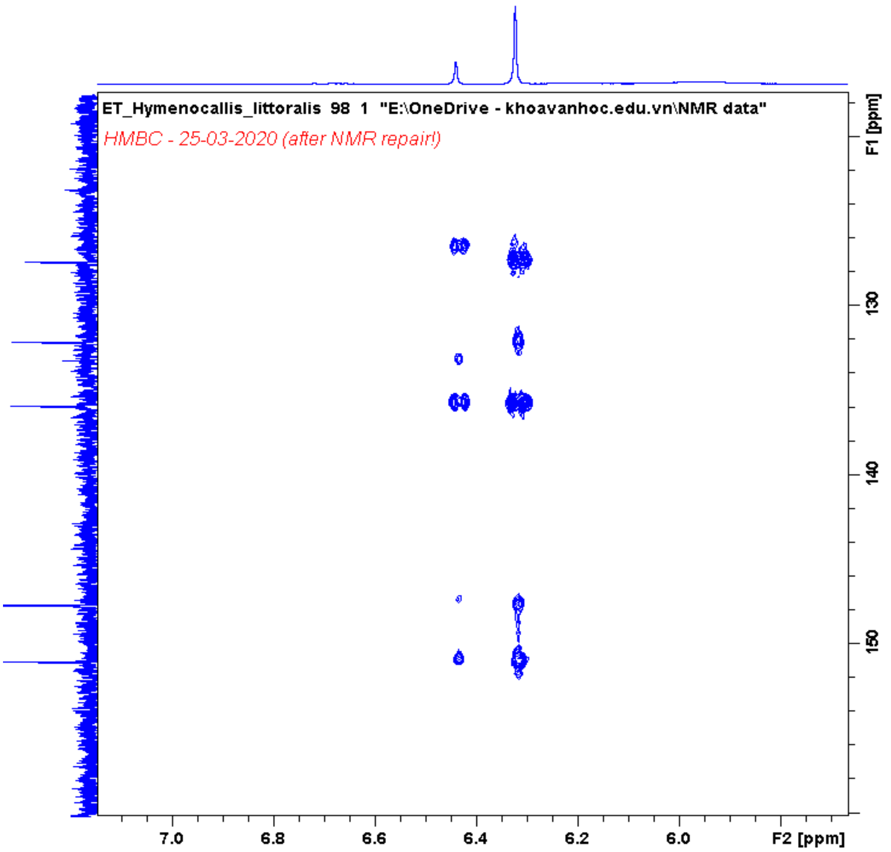


**Figure S7.** HMBC correlations of H-7 with the aromatic carbons.

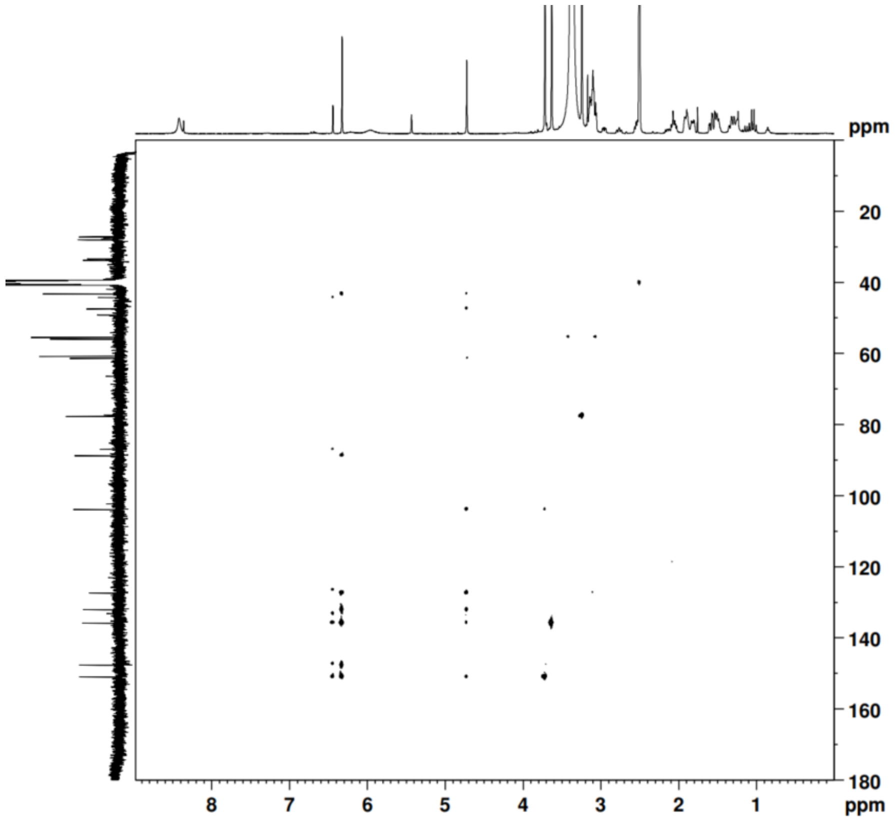


**Figure S8.** Long-range HMBC spectrum of compounds **1** and **2** in $(CD_3)_2SO$.

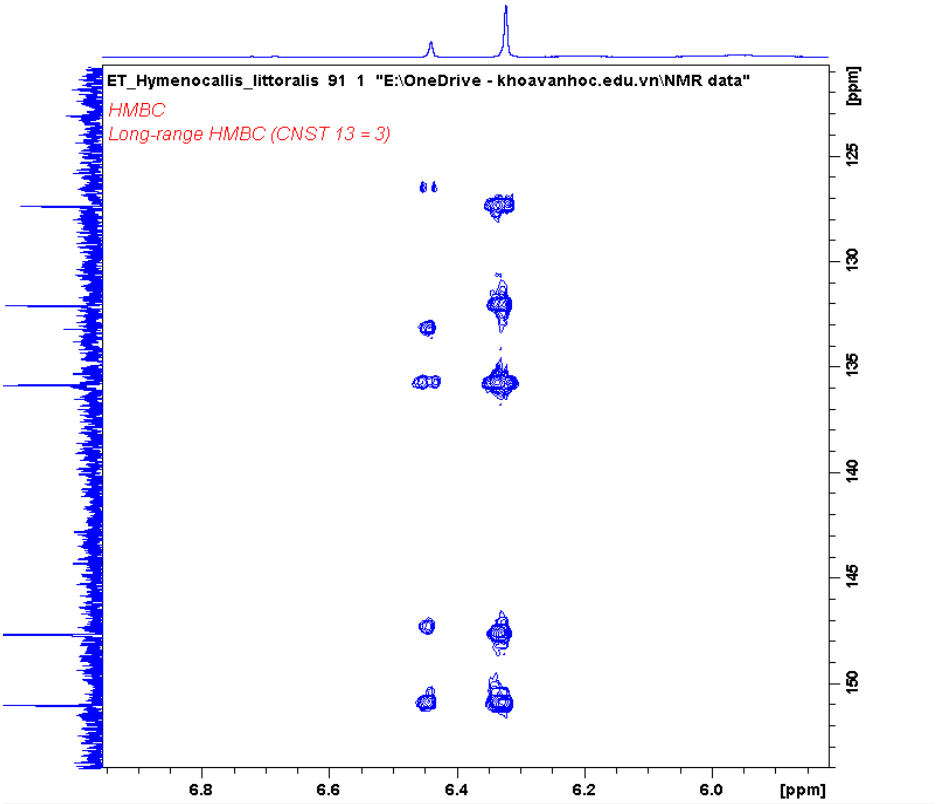


**Figure S9.** HMBC correlations of H-7 with the aromatic carbons (long-range HMBC).

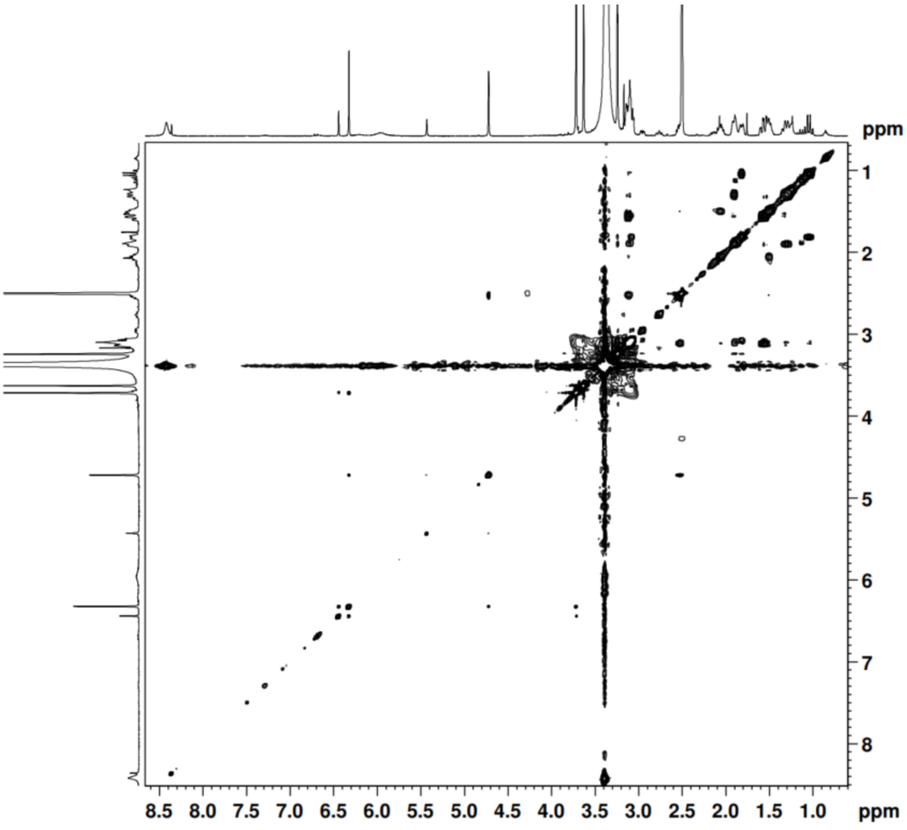


**Figure S10.** NOESY spectrum of compounds **1** and **2** in $(CD_3)_2SO$.

List of pulse sequences used in this study:

1. $^{1}H$: zg30, d1 = 2s, sw = 16.0203, o1p = 7.0 ppm (size of fid = 65536)
2. $^{13}C$: zgpg30, d1 = 2s, sw = 180 ppm, o1p = 90.0 (size of fid = 65536)
3. COSY: cosygpqf, d1 = 2s, sw = 10.0 x 10.0 ppm, o1p = 5.0 ppm, o2p = 5.0 ppm (size of fid: F2 – 2048, F1 – 128)
4. HSQC: hsqcetqpsi2, d1 = 2s, sw = 10.0 x 160.0 ppm, o1p = 5.0 ppm, o2p = 80.0 ppm (size of fid: F2 – 1024, F1 – 512)
5. NOESY: noesyphpr, d1 = 2s, d8 = 0.5s, PL9 = 60 dB (size of fid: F2– 2048, F1 – 256)
6. HMBC: hmbcgplpndqf, d1 = 2s, cnst13 = 10, sw = 10.0 x 220.0 ppm, o1p = 5.0 ppm, o2p = 110.0 ppm (size of fid: F2– 4096, F1 – 512)
7. Long-range HMBC acquisition parameters: hmbcgplpndqf, d1 = 2s, cnst13 = 3, sw = 10.0 x 220.0 ppm, o1p = 5.0 ppm, o2p = 110.0 ppm (size of fid: F2– 4096, F1 – 512)

**Table S1**. Experimental and computed NMR data (400 MHz, $(CD_3)_2SO$) of compound **1** (major epimer).

| | **Chemical shifts** | | **2D correlations** | | |
|---|---|---|---|---|---|
| Position | $\delta_C$, type | $\delta_H$ (*J* in Hz) | COSY | HMBC | NOESY |
| 1α | 27.1, $CH_2$ | 1.55 | H-1β | C-10b | H-2α |
| 1β | 27.1, $CH_2$ | 3.12* | H-1α | * | H-2β |
| 2α | 27.9, $CH_2$ | 1.90 m | H-2β | n.o. | H-1α |
| 2β | 27.9, $CH_2$ | 1.30 m | H-2α | n.o. | H-1β |
| 3 | 77.6, CH | 3.10* | | * | |
| 4α | 33.7, $CH_2$ | 1.82 m | H-4β | | |
| 4β | 33.7, $CH_2$ | 1.05 q (11.8) | H-4a, H-4α | C-4a, C-3 | |
| 4a | 61.3, CH | 3.07* | | C-10a, C-12 | |
| 6 | 88.8, CH | 4.72 s | | C-12, C-4a, C-7, C-10a, C-6a, C-12 (w), C-8 (w) | H-12b, H-7 |
| 6a | 132.0, C | - | | | |
| 7 | 103.8, CH | 6.32 s | | C-10b, C-6, C-10a, C-6a, C-9, C-10, C-8 | 8-OMe, H-6 |
| 8 | 150.9, C | - | | | |
| 9 | 135.8, C | - | | | |
| 10 | 147.7, C | - | | | |
| 10a | 127.3, C | - | | | |
| 10b | 43.3, C | - | | | |
| 11a | 33.3, $CH_2$ | 2.06 m | H-11b | C-10a | |
| 11b | 33.3, $CH_2$ | 1.51* | H-11a | * | |
| 12a | 47.3, $CH_2$ | 3.12* | H-12b | * | |
| 12b | 47.3, $CH_2$ | 2.52 | H-12a | n.o. | |
| 3-OMe | 55.3, $CH_3$ | 3.24 s | | C-3 | |
| 8-OMe | 55.9, $CH_3$ | 3.72 s | | C-8 | |
| 9-OMe | 60.6, $CH_3$ | 3.63 s | | C-9 | |

*overlapping signals, w = weak, n.o. = not observed

**Table S2**. Experimental and computed NMR data (400 MHz, $(CD_3)_2SO$) of compound **2** (minor epimer).

| | **Chemical shifts** | | **2D correlations** | | |
|---|---|---|---|---|---|
| Position | $\delta_C$, type | $\delta_H$ (*J* in Hz) | COSY | HMBC | NOESY |
| 1α | 27.1 | 1.55 | * | n.o. | |
| 1β | 27.1 | 3.12* | * | n.o. | |
| 2α | 27.9 | 1.90 m | * | n.o. | |
| 2β | 27.9 | 1.30 m | * | n.o. | |
| 3 | 77.3 | 3.10* | * | n.o. | |
| 4α | 33.9 | 1.88 m | H-4β | n.o. | |
| 4β | 33.9 | 1.13 q (11.8) | H-4α | n.o. | |
| 4a | 66.3 | 2.95 | H-4α, H-4β | n.o. | H-4α, H-6, H-1α (w) |
| 6 | 86.8 | 5.43 s | | n.o. | |
| 6a | 133.1 | - | | n.o. | |
| 7 | 102.3 | 6.44 s | | C-10b (long range HMBC), C-6, C-10a, C-6a, C-9, C-10, C-8 | 8-OMe |
| 8 | 150.8 | - | | | |
| 9 | 135.6 | - | | | |
| 10 | 147.4 | - | | | |
| 10a | 126.5 | - | | | |
| 10b | 44.3 | - | | | |
| 11a | 35.0 | 2.13 m | H-11b | n.o. | |
| 11b | 35.0 | 1.48* | H-11a | n.o. | |
| 12a | 41.9 | 3.17* | H-12b | n.o. | |
| 12b | 41.9 | 2.76 | H-11a, H-12a | n.o. | |
| 3-OMe | 55.3 | 3.24 s | | n.o. | |
| 8-OMe | 55.9 | 3.72 s | | n.o. | |
| 9-OMe | 60.6 | 3.63 s | | n.o. | |

*overlapping signals, w = weak, n.o. = not observed.

| Functional | Solvent? | Basis Set | Type of Data | | | | | |
|---|---|---|---|---|---|---|---|---|
| mPW1PW91 | PCM | 6-311+G(d,p) | Shielding Tensors | | | | | |
| | Isomer 1 | Isomer 2 | Isomer 3 | Isomer 4 | Isomer 5 | Isomer 6 | Isomer 7 | Isomer 8 |
| sDP4+ (H data) | 99.91% | 0.00% | 0.00% | 0.00% | 0.09% | 0.00% | 0.00% | 0.00% |
| sDP4+ (C data) | 94.45% | 0.00% | 0.00% | 0.00% | 5.54% | 0.00% | 0.00% | 0.00% |
| sDP4+ (all data) | 99.99% | 0.00% | 0.00% | 0.00% | 0.01% | 0.00% | 0.00% | 0.00% |
| uDP4+ (H data) | 98.29% | 0.07% | 0.00% | 0.00% | 1.63% | 0.00% | 0.00% | 0.00% |
| uDP4+ (C data) | 99.99% | 0.00% | 0.00% | 0.00% | 0.01% | 0.00% | 0.00% | 0.00% |
| uDP4+ (all data) | 100.00% | 0.00% | 0.00% | 0.00% | 0.00% | 0.00% | 0.00% | 0.00% |
| DP4+ (H data) | 100.00% | 0.00% | 0.00% | 0.00% | 0.00% | 0.00% | 0.00% | 0.00% |
| DP4+ (C data) | 100.00% | 0.00% | 0.00% | 0.00% | 0.00% | 0.00% | 0.00% | 0.00% |
| DP4+ (all data) | 100.00% | 0.00% | 0.00% | 0.00% | 0.00% | 0.00% | 0.00% | 0.00% |

**Figure S11.** DP4+ probability of compound **1** (the major epimer of 6-hydroxyhippeastidine).

| Functional | Solvent? | Basis Set | Type of Data | | | | | |
|---|---|---|---|---|---|---|---|---|
| mPW1PW91 | PCM | 6-311+G(d,p) | Shielding Tensors | | | | | |
| | Isomer 1 | Isomer 2 | Isomer 3 | Isomer 4 | Isomer 5 | Isomer 6 | Isomer 7 | Isomer 8 |
| sDP4+ (H data) | 0.00% | 100.00% | 0.00% | 0.00% | 0.00% | 0.00% | 0.00% | 0.00% |
| sDP4+ (C data) | 0.09% | 96.03% | 0.00% | 0.00% | 0.00% | 3.88% | 0.00% | 0.00% |
| sDP4+ (all data) | 0.00% | 100.00% | 0.00% | 0.00% | 0.00% | 0.00% | 0.00% | 0.00% |
| uDP4+ (H data) | 8.42% | 91.21% | 0.00% | 0.00% | 0.12% | 0.24% | 0.00% | 0.00% |
| uDP4+ (C data) | 0.30% | 99.70% | 0.00% | 0.00% | 0.00% | 0.01% | 0.00% | 0.00% |
| uDP4+ (all data) | 0.03% | 99.97% | 0.00% | 0.00% | 0.00% | 0.00% | 0.00% | 0.00% |
| DP4+ (H data) | 0.00% | 100.00% | 0.00% | 0.00% | 0.00% | 0.00% | 0.00% | 0.00% |
| DP4+ (C data) | 0.00% | 100.00% | 0.00% | 0.00% | 0.00% | 0.00% | 0.00% | 0.00% |
| DP4+ (all data) | 0.00% | 100.00% | 0.00% | 0.00% | 0.00% | 0.00% | 0.00% | 0.00% |

**Figure S12.** DP4+ probability of compound **2** (the minor epimer of 6-hydroxyhippeastidine).
